# Carbon and nitrogen partitioning of transgenic rice T2A-1 (Cry2A*) with different nitrogen treatments

**DOI:** 10.1038/s41598-019-41267-1

**Published:** 2019-03-29

**Authors:** Lin Ling, Xuexue Li, Kangxu Wang, Mingli Cai, Yang Jiang, Cougui Cao

**Affiliations:** Ministry of Agriculture Key Laboratory of Crop Physiology, Ecology and Cultivation (The Middle Reaches of Yangtze River), Huazhong Agricultural University, Wuhan, 430070 P. R. China

## Abstract

Nitrogen (N) and carbon(C) metabolisms in plants were investigated to assess different responses of Bt and non-Bt rice to different N treatments. T2A-1 (Bt rice variety) inserted with Cry2A* protein to resist Lepidoptera and its parental line MH63 was adopted in this study. The total N accumulation presented no statistical difference. But nitrogen contents in different parts of rice plant were significantly different between the two lines, especially on leaf and spike part. This study revealed that the nitrogen in leaf of T2A-1 was far more than that of MH63; however, the nitrogen in spike of T2A-1 was less than that of MH63. In addition, MH63 assimilated more carbon than T2A-1. However, the distribution proportion of carbon in leaf, stem and spike of T2A-1 and MH63 were both 1:1:1. What’s more, our study of the difference in metabolism pathway based on proteomics analysis provided more insights on the responses of two lines of Bt and non-Bt rice to different N treatments. And amino acid metabolism, energy metabolism, and carbohydrate metabolism presented significant difference between two lines. In addition, the number of differentially expressed proteins with N deficiency treatment was almost twice as many as that with normal N treatment. It could be inferred that the insertion of Cry2A* in T2A-1 may bring about effects on carbon and nitrogen allocation and related metabolisms, especially under N deficiency environment.

## Introduction

Since rice yield suffers great loss due to lepidopteran pests^1^, scientists worked on possible control measures including developing transgenic *Bt* rice in the last few decades^2–7^. Cultivation area of transgenic Bt crop was up to 98.5 million hectares worldwide in 2016^8^. In recent years, planting Bt crops has been widely recognized as a pest prevention measure. Although many *Bt* genes have been found, only a few of them were selected for developing transgenic crops. *Cry2A* protein was reported to have different binding regions in brush border membrane vesicles and to present no cross-resistance^9–12^. *Cry2A* gene could be applied to develop marker-free *Bt* transgenic rice as a resistance source of gene pyramid. As a result, the evolution of insects’ resistance to toxicity of Bt rice would be postponed. Thus, *Cry2A* was considered as an important protein for bio-breeding scientists. However, several previous studies demonstrated the differences in the agronomy traits and physiological metabolism in some rice lines inserted by exogenous insect-resistant genes^13–19^. These differences included reduced plant heights and root lengths^14^, fewer grains per panicle^17^, decreased setting rates^5,14,17,18,20^, and so on, which in turn led to reductions in grain yield. The obvious advantage of Bt rice in pest resistance does not balance out its unintended effects resulting in lower yield^21^. These unintended effects brought by transgenic crop were supposed to have three sources: the disruption of endogenous genetic background^22^, the somaclonal variation happened during tissue culture processes^23^, and “bio-burden” brought by additional transgenic protein synthesis process^24^. “Bio-burden” was described as burden on material and energy, which could influence N and C metabolisms in plant.

The N amount demanded by plants is highest among all mineral nutrients. And it was one of the most important elements influencing crop yield directly. Generally, with the increase of N application, more *Bt* protein in plant tissues could be detected^20,25,26^. According to *Bruns* and *Abel*^25^, there was no significant difference in the total N uptake between Bt maize and their non-Bt counterparts. However, some other Bt hybrid maize strains were demonstrated to have lower N accumulation in grains but higher N accumulation in straws compared with their non-Bt control lines^27^. Moreover, Pioneer 38W36Bt, a Bt hybrid maize, accumulated more N in its kernels and entire maize plant than its conventional control Pioneer 38W36. But the two maize lines showed similar N and leaf chlorophyll contents at the late growth stages^28^. Besides, Bt cotton was reported to have higher N content and N metabolism enzymes than its non-Bt counterpart^29^. While, relatively less reports have been made about N metabolism in Bt rice. Thus, studies on responses of Bt rice lines to different N treatments are of great necessity.

Gurr and Rushton (2005) proposed that the difference between transgenic and non-transgenic lines might have been brought about by extra consumption of material and energy to maintain exogenous metabolism in plants^24^. Based on their hypothesis, C and N metabolism in transgenic plants were likely to change compared with their non-transgenic counterparts. C and N contents and ratio of C and N were assumed to be the indicators representing C and N metabolism in plants^30^. Therefore, whether the distribution and allocation of C and N in transgenic Cry2A* rice are different from those of its parent line could provide essential evidence for Bt rice breeding. This study was aimed to (1) explore the distribution and allocation of C and N in transgenic Cry2A* rice (T2A-1) and its conventional (non-transgenic) parent line MH63, a widely used indica cytoplasm male sterile (CMS) restorer rice line in Asia^5^; (2) attempt to discover the differences of proteins expression related to C and N metabolism under different N treatments combining physiological indicators of C and N metabolism with proteomics analysis.

## Results

### Biomass accumulation and distribution in Bt (T2A-1) and non-Bt rice

Significant differences in dry matter of each part including stem, leaf and spike were observed between plants of MH63 and T2A-1(Table 1) under different nitrogen treatments. And dry matter of each part increased with the addition of nitrogen fertilizer under several treatments. Meanwhile, the total biomass of the two lines showed no significant difference under each N application treatment. For example, at the FS stage, the dry weight of the stem of T2A-1 was 21.2% lower than that of MH63 under N1 treatment, and the biomass of the spike of T2A-1 were 31.4% and 30.7% lower than that of MH63 under N3 and N4 treatments, respectively. What’s more, on day 15 after FS, similar results were observed in spike under N2, N3 and N4 treatments, while the opposite results were observed in leaf under the same treatments.

**Table 1:** Biomass and spike weight of T2A-1 and MH63 under different nitrogen treatment at several growth periods (g).

N treatment	Variety	Biomass	FL			15 days after FL			
			Stem	Leaf	Spike	Biomass	Stem	Leaf	Spike
N1	T2A-1	49.02 a ± 2.77	32.08 a ± 2.31	11.52 a ± 0.33	5.42 a ± 0.32	62.90 a ± 3.13	45.31 a ± 3.28	9.31 a ± 0.39	8.29 a ± 0.32
	MH63	45.01 a ± 3.56	28.69 a ± 2.35	10.14 a ± 0.78	6.18 a ± 0.59	67.23 a ± 5.66	48.28 a ± 4.33	10.10 a ± 0.58	8.86 a ± 0.82
N2	T2A-1	45.77 a ± 2.59	30.19 a ± 2.13	11.29 a ± 0.25	4.29 b ± 0.36	73.28 a ± 3.79	54.52 a ± 2.12	10.25 a ± 0.55	8.51 a ± 1.39
	MH63	52.69 a ± 3.24	33.93 a ± 2.35	12.44 a ± 0.55	6.31 a ± 0.54	69.24 a ± 2.74	49.76 a ± 2.03	11.39 a ± 0.82	8.09 a ± 1.38
N3	T2A-1	51.70 a ± 1.92	33.34 a ± 1.27	12.66 a ± 0.29	5.70 a ± 0.59	70.82 a ± 1.90	48.37 a ± 2.25	13.50 a ± 1.18	8.95 a ± 0.53
	MH63	54.18 a ± 4.71	33.77 a ± 3.35	12.97 a ± 0.59	7.44 a ± 0.96	59.87 a ± 5.29	38.86 a ± 3.72	10.25 a ± 0.91	10.76 a ± 0.86
N4	T2A-1	59.88 a ± 2.70	38.28 a ± 2.65	16.72 a ± 0.57	4.87 b ± 0.34	89.99 a ± 3.98	59.88 a ± 4.28	18.02 a ± 0.24	12.09 a ± 1.24
	MH63	57.67 a ± 5.66	34.47 a ± 5.00	15.37 a ± 0.36	7.83 a ± 0.44	86.14 a ± 7.20	56.90 a ± 5.41	14.13 b ± 1.10	15.11 a ± 0.79
Mean	T2A-1	51.59	33.47	13.05	5.06	74.25	52.02	12.77	9.46
	MH63	52.39	32.72	12.73	6.94	71.36	48.45	11.47	10.71
**Analysis of variance**									
N level		*	**	**	NS	**	**	**	**
Genotype		NS	NS	NS	**	NS	NS	**	NS
N level × Genotype		NS	NS	NS	NS	NS	NS	**	NS

Data are presented as the means ± standard deviation (SD, n = 3). Mean were the mean weights of T2A-1 and MH63 under the four N treatments. Lowercase letters indicate SNK variance between groups under same nitrogen treatments at same sampling time. The * indicate a significant source of variance at P = 0.05, while ** at P = 0.01. NS means no significance. N1: 0.2 g N pot^−1^; N2: 0.35 g N pot^−1^; N3: 0.5 g N pot^−1^; N4: 1 g N pot^−1^. FS: flowering stage.

### Carbon accumulation and distribution in Bt (T2A-1) and non-Bt rice

At two sampling stages, significant difference in carbon content of each part including stem, leaf and spike was found between the T2A-1 and MH63, and it was true with the total carbon content of the aboveground (Table 2). While, in flowering stage (FS), the mean carbon accumulation of the aboveground in T2A-1 was 610.6 mg under the four treatments N1–4, which was 221.6 mg lower than that of MH63. On day 15 after FS, this difference reached up to 447.9 mg. However, T2A-1 was similar to MH63 in the distribution of carbon in three parts (leaf, stem, spike) of the plant with the distribution ratio being 1:1:1.

**Table 2:** Accumulation and distribution of C of T2A-1 and MH63 under different nitrogen treatments at several growth periods (mg).

N treatment	Variety	FS				15 days after FS			
		Aboveground	Stem	Leaf	Spike	Aboveground	Stem	Leaf	Spike
N1	T2A-1	644.8 a ± 36.6	206.9 a ± 12.0	214.5 a ± 11.8	223.5 a ± 12.9	1045.8 a ± 140.4	356.1 a ± 47.6	363.7 a ± 46.4	326.0 a ± 46.6
	MH63	737.7 a ± 70.7	233.4 a ± 22.2	245.5 a ± 24.7	258.7 a ± 25.0	1112.4 a ± 82.8	375.1 a ± 26.3	382.5 a ± 27.9	354.9 a ± 29.2
N2	T2A-1	516.4 b ± 45.7	169.0 a ± 15.8	169.2 b ± 13.6	178.2 b ± 16.3	1082.2 a ± 53.3	365.3 a ± 19.3	376.0 a ± 19.4	340.9 a ± 14.8
	MH63	758.3 a ± 66.0	243.9 a ± 22.2	251.5 a ± 21.6	262.9 a ± 22.6	1373.5 a ± 101.0	463.2 a ± 32.45	468.9 a ± 33.2	441.4 a ± 35.4
N3	T2A-1	686.3 a ± 70.2	220.3 a ± 23.5	228.0 a ± 22.7	238.0 a ± 24.0	1069.7 b ± 76.4	352.6 b ± 25.4	371.6 b ± 26.5	345.5 b ± 24.6
	MH63	897.5 a ± 116.2	288.5 a ± 37.3	302.2 a ± 39.8	306.8 a ± 39.2	2100.7 a ± 127.6	694.8 a ± 38.8	718.4 a ± 41.6	687.5 a ± 47.3
N4	T2A-1	594.8 b ± 38.9	187.9 b ± 9.0	200.8 b ± 14.4	206.2 b ± 15.9	1515.7 a ± 67.6	500.2 a ± 18.2	524.7 a ± 25.8	490.8 a ± 23.7
	MH63	935.3 a ± 55.8	299.7 a ± 16.8	317.3 a ± 19.6	318.4 a ± 19.4	1918.3 a ± 150.6	636.0 a ± 47.5	651.1 a ± 48.9	631.2 a ± 54.8
Mean	T2A-1	610.6	196.0	203.1	211.5	1178.4	393.6	409.0	375.8
	MH63	832.2	266.4	279.1	286.7	1626.2	442.3	555.2	528.8
**Analysis of variance**									
N level		NS	NS	NS	NS	NS	**	**	**
Genotype		**	**	**	**	**	**	**	**
N level × Genotype		NS	NS	NS	NS	**	**	**	**

Data are presented as the means ± standard deviation (SD, n = 3). Lowercase letters indicate SNK variance between groups under same nitrogen treatments at same sampling time. The * indicate a significant source of variance at P = 0.05, while ** at P = 0.01. NS means no significance. N1: 0.2 g N pot^−1^; N2: 0.35 g N pot^−1^; N3: 0.5 g N pot^−1^; N4: 1 g N pot^−1^. FS: flowering stage.

### Nitrogen accumulation and distribution in Bt (T2A-1) and non-Bt rice

There was no significant difference in nitrogen assimilation between *Bt* (T2A-1) and non-*Bt* rice. However, the distribution of nitrogen in leaf, stem and spike presented several differences at the two sampling times respectively on FS and day 15 after FS, especially on leaf and spike part (Table 3). To be more specific, mean value of nitrogen concentration in leaf of T2A-1 were 6.41% and 6.35% higher than that of MH63 respectively (Table 4). While the mean value in spike of T2A-1 were 4.74% and 8.38% lower than that of MH63. And the nitrogen content in each part of both lines T2A-1 and MH63 exhibited the same ranking, namely, leaf > stem > spike at FS. Whereas 15 days after FS, the ranking of the two lines was changed into stem > leaf > spike.

**Table 3:** Accumulation and distribution of N of T2A-1 and MH63 under different nitrogen treatments at several growth periods (mg).

N treatment	Variety	FS				15 days after FS			
		Aboveground	Stem	Leaf	Spike	Aboveground	Stem	Leaf	Spike
N1	T2A-1	36.7 a ± 1.0	11.7 b ± 0.7	19.6 a ± 0.1	5.4 a ± 0.4	39.4 a ± 2.0	16.8 a ± 1.6	14.1 a ± 0.2	8.5 a ± 0.9
	MH63	37.0 a ± 3.3	14.9 a ± 0.2	16.3 a ± 1.3	7.2 a ± 1.2	41.2 a ± 0.6	18.2 a ± 1.07	12.6 a ± 0.9	10.4 a ± 0.4
N2	T2A-1	39.6 a ± 0.4	14.3 a ± 0.3	20.7 a ± 0.5	4.6 a ± 0.5	46.4 a ± 1.9	19.2 a ± 1.36	17.7 a ± 1.5	9.5 b ± 0.4
	MH63	45.4 a ± 2.3	16.2 a ± 0.7	21.9 a ± 1.0	7.4 a ± 1.1	49.9 a ± 2.2	21.9 a ± 1.6	14.9 a ± 0.1	13.2 a ± 0.7
N3	T2A-1	50.6 a ± 1.1	16.5 a ± 0.2	26.4 a ± 0.9	7.8 b ± 0.4	52.1 a ± 3.4	21.3 a ± 1.1	18.9 a ± 0.5	11.3 b ± 0.5
	MH63	51.5 a ± 4.6	16.6 a ± 2.4	24.7 a ± 1.5	11.4 a ± 0.1	59.2 a ± 1.7	28.1 a ± 1.5	15.1 b ± 0.9	22.4 a ± 1.5
N4	T2A-1	70.6 a ± 1.5	24.0 a ± 2.4	39.1 a ± 0.6	7.5 b ± 0.5	71.9 a ± 5.6	31.7 a ± 1.07	23.2 a ± 4.4	17.0 b ± 0.5
	MH63	72.4 a ± 1.1	25.4 a ± 1.1	36.2 a ± 1.4	10.8 a ± 0.8	73.5 a ± 24	28.1 a ± 1.46	23.4 a ± 1.5	24.5 a ± 0.9
Mean	T2A-1	49.4	16.6	26.5	6.3	52.5	22.3	18.5	11.6
	MH63	51.6	18.3	24.8	9.2	56.0	24.1	16.5	17.6
**Analysis of variance**									
N level		**	**	**	**	**	**	**	**
Genotype		NS	NS	*	**	*	NS	NS	**
N level × Genotype		NS	NS	NS	NS	NS	NS	NS	**

Data are presented as the means ± standard deviation (SD, n = 3). Lowercase letters indicate SNK variance between groups under same nitrogen treatments at same sampling time. The * indicate a significant source of variance at P = 0.05, while ** at P = 0.01. NS means no significance. N1: 0.2 g N pot^−1^; N2: 0.35 g N pot^−1^; N3: 0.5 g N pot^−1^; N4: 1 g N pot^−1^. FS: flowering stage.

**Table 4:** Distribution of N of T2A-1 and MH63 under different nitrogen treatments at several growth periods (%).

N treatment	Variety	FS				15 days after FS			
		Aboveground	Stem	Leaf	Spike	Aboveground	Stem	Leaf	Spike
N1	T2A-1	100 ± 0.00	31.92 b ± 1.17	53.41 a ± 1.16	14.67 a ± 0.96	100 ± 0.00	42.52 a ± 2.43	35.85 a ± 1.87	21.63 a ± 2.10
	MH63	100 ± 0.00	39.05 a ± 2.14	42.45 b ± 2.05	18.50 a ± 2.33	100 ± 0.00	44.11 a ± 1.92	30.72 a ± 2.54	25.17 a ± 0.65
N2	T2A-1	100 ± 0.00	36.05 a ± 0.60	52.27 a ± 0.77	11.68 a ± 1.19	100 ± 0.00	41.45 a ± 2.76	38.15 a ± 2.26	20.41 b ± 0.68
	MH63	100 ± 0.00	35.79 a ± 1.55	48.12 b ± 0.34	16.08 a ± 1.87	100 ± 0.00	43.73 a ± 1.44	29.95 b ± 1.30	26.32 a ± 0.85
N3	T2A-1	100 ± 0.00	32.52 a ± 0.93	52.08 a ± 0.85	15.40 b ± 0.63	100 ± 0.00	41.28 a ± 1.72	36.66 a ± 0.17	22.05 b ± 1.56
	MH63	100 ± 0.00	31.25 a ± 2.01	46.91 b ± 0.59	21.84 a ± 1.42	100 ± 0.00	36.66 a ± 0.84	25.56 b ± 2.14	37.77 a ± 1.46
N4	T2A-1	100 ± 0.00	33.96 a ± 2.64	55.46 a ± 1.96	10.58 b ± 0.86	100 ± 0.00	44.41 a ± 2.12	31.69 a ± 3.96	23.89 b ± 1.92
	MH63	100 ± 0.00	35.08 a ± 1.14	50.07 a ± 2.04	14.86 a ± 0.99	100 ± 0.00	37.03 a ± 1.78	30.74 a ± 1.25	32.23 a ± 0.62
Mean	T2A-1	100	33.61	53.30	13.08	100	42.42	35.59	21.99
	MH63	100	35.29	46.89	17.82	100	40.38	29.24	30.37
**Analysis of variance**									
N level		—	NS	*	**	—	NS	NS	**
Genotype		—	NS	**	**	—	NS	**	**
N level × Genotype		—	**			—	NS	NS	**

Data are presented as the means ± standard deviation (SD, n = 3). Lowercase letters indicate SNK variance between groups under same nitrogen treatments at same sampling time. The * indicate a significant source of variance at P = 0.05, while ** at P = 0.01. NS means no significance. N1: 0.2 g N pot^−1^; N2: 0.35 g N pot^−1^; N3: 0.5 g N pot^−1^; N4: 1 g N pot^−1^. FS: flowering stage.

### C/N ratio in different part of Bt (T2A-1) and non-Bt rice

Under the four nitrogen treatments, C/N ratio in different parts of T2A-1 and MH63 presented significant difference (Table 5). At both sampling time, i.e., FS and day 15 after FS, C/N ratio was the highest in stem, and the lowest in leaf. Furthermore, C/N ratio of T2A-1 was significantly lower under N4 treatment than under other N treatments. And the C/N ratio of MH63 didn’t show obvious difference under its four treatments. At FS, C/N ratio in stem of T2A-1 is higher under N1 treatment than under N2 and N3 treatments. While, on day 15 after FS, there was no difference on C/N ratio under all treatments N1–4 of T2A-1. And C/N ratio in leaf and spike was the lowest under N4 treatment, which was significantly lower than that under N1 treatment. The C/N ratio of whole aboveground part of T2A-1 was significantly lower than that of MH63, and so was the C/N ratio in leaf.

**Table 5:** C/N of T2A-1 and MH63 under different nitrogen treatments at several growth periods.

N treatment	Variety	FS				15 days after FS			
		Aboveground	Stem	Leaf	Spike	Aboveground	Stem	Leaf	Spike
N1	T2A-1	17.56 b ± 0.69	104.38 a ± 3.00	23.31 a ± 0.46	41.62 a ± 0.97	26.43 a ± 2.65	111.34 a ± 10.56	27.46 a ± 1.10	38.09 a ± 1.74
	MH63	19.94 a ± 0.39	80.59 b ± 2.55	24.70 a ± 0.34	37.05 a ± 2.95	26.97 a ± 1.61	97.32 a ± 4.44	29.25 a ± 1.22	34.10 a ± 1.52
N2	T2A-1	13.04 a ± 1.22	83.38 a ± 7.51	21.53 a ± 0.63	38.56 a ± 0.28	23.30 b ± 0.20	112.43 a ± 2.12	24.68 a ± 1.50	36.07 a ± 1.36
	MH63	16.64 a ± 0.71	80.69 a ± 3.64	22.69 a ± 0.10	36.59 a ± 3.12	27.44 a ± 0.80	91.63 b ± 5.98	25.46 a ± 0.88	33.53 a ± 1.30
N3	T2A-1	13.53 a ± 1.25	78.31 a ± 2.68	19.25 b ± 0.51	30.35 a ± 1.54	20.78 b ± 2.27	101.48 a ± 13.51	24.22 b ± 0.32	30.49 a ± 1.18
	MH63	17.43 a ± 1.85	80.37 a ± 8.71	21.39 a ± 0.48	29.90 a ± 0.55	35.42 a ± 1.50	98.38 a ± 5.57	27.74 a ± 0.25	30.67 a ± 0.84
N4	T2A-1	8.44 b ± 0.63	62.15 a ± 4.41	17.64 a ± 0.52	27.64 a ± 0.18	21.44 a ± 2.40	74.00 a ± 4.73	19.69 a ± 0.95	28.91 a ± 0.93
	MH63	12.91 a ± 0.66	51.76 a ± 6.26	17.28 a ± 1.14	29.67 a ± 0.92	26.07 a ± 1.56	79.94 a ± 7.64	23.40 a ± 1.45	28.96 a ± 1.05
Mean	T2A-1	13.14	82.06	20.43	34.54	22.99	99.81	24.01	33.41
	MH63	16.73	73.35	21.52	33.30	28.98	91.82	26.46	31.82
**Analysis of variance**									
N level		**	**	**	**	NS	**	**	**
Genotype		**	*	**	NS	**	NS	**	NS
N level × Genotype		NS	NS	NS	NS	**	NS	NS	NS

Data are presented as the means ± standard deviation (SD, n = 3). Lowercase letters indicate SNK variance between groups under same nitrogen treatments at same sampling time. The * indicate a significant source of variance at P = 0.05, while ** at P = 0.01. NS means no significance. N1: 0.2 g N pot^−1^; N2: 0.35 g N pot^−1^; N3: 0.5 g N pot^−1^; N4: 1 g N pot^−1^. FS: flowering stage.

### Differentially expressed proteins related to C and N metabolism in leaves of MH63 and T2A-1 by iTRAQ

**Figure 1:**
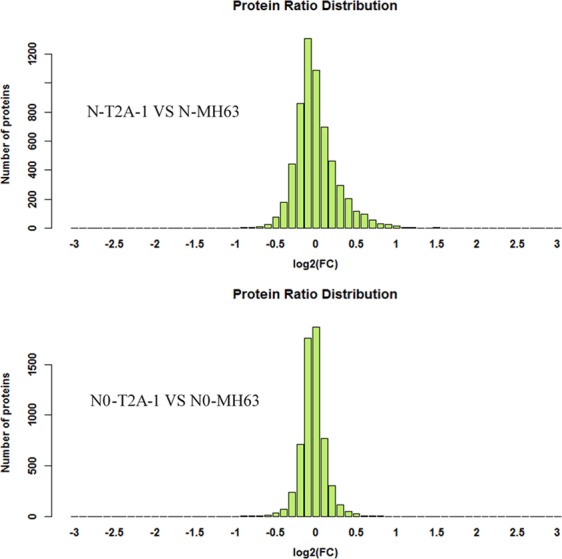
Protein ratio distribution of the two comparison groups: N-T2A-1 vs N-MH63 and N0-T2A-1 vs N0-MH63.

**Figure 2:**
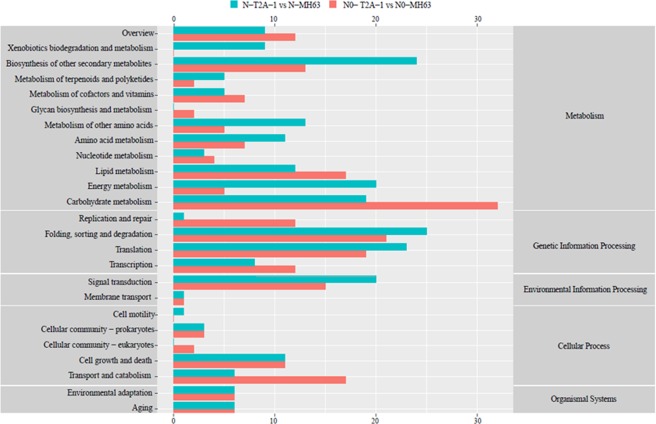
KEGG pathway variance distribution of the two comparison groups: N-T2A-1 vs N-MH63 and N0-T2A-1 vs N0-MH63.

Flag leaves of MH63 and T2A-1 under RN(1 g N·plant^−1^) and N0 (0 g N·plant^−1^) treatments were collected for proteomics analysis in iTRAQ (isobaric tags for relative and absolute quantification) method. The analysis results indicated that of a total of 6040 proteins identified in the experiment, 206 proteins were up-regulated and 315 proteins were down-regulated under RN treatment (Fig. 1). While 320 up-regulated and 217 down-regulated proteins were identified in the leaves of two rice lines at N0 level. In addition, the number of those differentially expressed proteins related to C and N metabolism under the two N treatments were 55 and 61 (Tables 6 and 7), occupying 17.2% and 28.1% of the total difference at RN and N0 levels respectively, and the KEGG analysis of those differentially expressed proteins were shown in Fig. 2.

**Table 6:** Differentially expressed proteins of T2A-1 compared with MH63 under RN treatment.

Description	Accession	Coverage (%)	Unique Peptides	Peptides	average N-LL/N-MH63	Significance A
Putative uncharacterized protein	B8AJE7	35.67	2	13	2.017	0.000
1-aminocyclopropane-1-carboxylate oxidase	O65031	8.41	2	2	1.995	0.000
Putative uncharacterized protein	B8B0S8	2.73	1	1	1.883	0.001
Os01g0832000 protein	Q0JI11	2.85	1	1	1.868	0.001
1,2-dihydroxy-3-keto-5-methylthiopentene dioxygenase	A2Z7C5	5.43	1	1	1.821	0.002
Os06g0570566 protein	A0A0P0WY46	7.69	1	1	1.815	0.002
Os02g0119800 protein	Q0E4H0	3.08	1	1	1.799	0.002
Uncharacterized protein	A2Z839	4.98	1	1	1.787	0.002
Amine oxidase	B8B7J8	2.84	1	1	1.755	0.003
Putative uncharacterized protein	B8B9C4	55.4	1	15	1.719	0.004
Hexokinase-7	Q1WM16	8.42	2	3	1.660	0.007
Os08g0501132 protein (Fragment)	A0A0P0XHL9	8.6	1	1	1.636	0.009
Putative uncharacterized protein	B8A7D9	7.14	1	1	1.627	0.010
Putative uncharacterized protein	B8BJ02	10.06	1	1	1.622	0.011
Hydroxycinnamoyltransferase 1	Q0JBZ8	6.11	2	2	1.621	0.011
Os02g0175600 protein	Q6EUQ7	24.31	1	3	1.616	0.011
Lipoxygenase	A3AU93	4.59	3	3	1.610	0.012
Putative uncharacterized protein	B8AFI3	2.1	1	1	1.610	0.012
UDP-glucose 6-dehydrogenase 4	Q2QS14	44.58	1	16	1.585	0.015
Putative uncharacterized protein	B8ADS0	2.98	1	1	1.573	0.017
Os05g0301700 protein	Q0DJC3	36.18	1	6	1.565	0.018
Putative uncharacterized protein	B8B9C5	54.35	1	15	1.560	0.019
Beta-amylase	A3ADZ2	2.52	1	1	1.545	0.022
Putative uncharacterized protein	A2WV09	4.46	1	1	1.521	0.027
Putative uncharacterized protein	B8B184	3.58	1	1	1.520	0.027
Pyruvate kinase 2, cytosolic	Q2QXR8	37	1	17	1.518	0.028
Peroxisomal (S)-2-hydroxy-acid oxidase GLO3	B8AUI3	10.35	2	4	1.516	0.029
Os08g0495800 protein	Q6Z5C3	2.25	1	1	1.503	0.032
60 S ribosomal protein L27	A2ZAB3	38.24	3	6	1.467	0.044
Peroxidase	A3AB79	1.61	1	1	0.572	0.000
Ribulose bisphosphate carboxylase large chain (Fragment)	Q5K3B1	64.71	2	37	0.599	0.000
30 S ribosomal protein S16, chloroplastic	A0A173CU41	28.57	2	2	0.628	0.000
Os03g0283600 protein (Fragment)	A0A0P0VW68	4.59	1	1	0.634	0.000
Putative uncharacterized protein	B8ARH5	4.64	1	1	0.645	0.000
3-oxoacyl-[acyl-carrier-protein] synthase	A3AU82	21.32	2	6	0.679	0.002
Putative uncharacterized protein	B8AWK7	20.51	1	12	0.686	0.002
Probable pyridoxal 5’-phosphate synthase subunit PDX1.2	Q8W3D0	35.78	1	10	0.702	0.004
Os02g0798100 protein	Q69QZ8	2.51	1	1	0.708	0.005
UDP-glucose 6-dehydrogenase 5	Q2QS13	41.46	2	16	0.712	0.006
Uncharacterized protein	B9F3L6	0.73	1	1	0.723	0.009
Beta-glucosidase 27	Q84YK7	3.81	2	2	0.728	0.010
Cytochrome c oxidase subunit 6b	Q9FE02	12.99	1	1	0.731	0.011
Inositol-3-phosphate synthase	O64437	13.92	4	4	0.732	0.012
Os03g0238600 protein	Q10PD0	4.13	1	1	0.733	0.012
Uncharacterized protein	B9FFK4	5.32	1	1	0.736	0.014
50 S ribosomal protein L35	Q67W51	4.11	1	1	0.748	0.019
Os01g0717000 protein (Fragment)	A0A0P0V7D0	9.09	1	1	0.751	0.020
ATP-dependent 6-phosphofructokinase	A2ZQ69	12.33	1	5	0.757	0.024
Glutathione peroxidase	B7FAE9	30.34	1	6	0.765	0.030
Cytochrome c	A2Y4S9	20.54	2	2	0.766	0.031
Putative 60 S ribosomal protein L24	Q8L3Y6	33.75	1	6	0.770	0.034
Os07g0613200 protein	Q8GSE9	35.62	4	4	0.771	0.035
60 S ribosomal protein L21, putative, expressed	Q10RZ3	20.73	1	5	0.772	0.036
UDP-glucose 6-dehydrogenase 3	Q9AUV6	63.96	4	21	0.772	0.036
Os12g0541000 protein	Q2QP59	11.71	3	3	0.775	0.039

**Table 7:** Differentially expressed proteins of T2A-1 compared with MH63 under N0 treatment.

Description	Accession	Coverage (%)	Unique Peptides	Peptides	average NO-LL/NO-MH63	Significance A
Hydroxycinnamoyltransferase 1	Q0JBZ8	6.11	2	2	1.609	0.000
Cytochrome P450 84A1, putative, expressed	Q109F2	23.58	4	9	1.509	0.000
UDP-glucose 6-dehydrogenase 4	Q2QS14	44.58	1	16	1.424	0.000
Os06g0570566 protein	A0A0P0WY46	7.69	1	1	1.402	0.000
Glutathione s-transferase (Fragment)	Q8L6H9	36.76	5	6	1.398	0.000
1,2-dihydroxy-3-keto-5-methylthiopentene dioxygenase	A2Z7C5	5.43	1	1	1.388	0.000
Phenylalanine ammonia-lyase	Q6K6Q1	27.44	6	15	1.378	0.000
Phenylalanine ammonia-lyase	P14717	35.81	16	20	1.374	0.000
Putative uncharacterized protein	B8B184	3.58	1	1	1.366	0.000
Peroxisomal (S)-2-hydroxy-acid oxidase GLO3	B8AUI3	10.35	2	4	1.364	0.001
Lipoxygenase	A3BUP8	34.27	13	24	1.336	0.001
Amine oxidase	B8B7J8	2.84	1	1	1.323	0.002
Phenylalanine ammonia-lyase	Q75HQ7	22.63	5	13	1.313	0.002
Phospho-2-dehydro-3-deoxyheptonate aldolase	Q0D4J5	13.04	5	6	1.312	0.002
Glycosyltransferase	A2XWB5	8.42	2	2	1.311	0.002
Tau class GST protein 4	Q6WSC3	27.62	6	7	1.290	0.004
Pyruvate kinase 2, cytosolic	Q2QXR8	37	1	17	1.282	0.006
Putative uncharacterized protein	B8B9C4	55.4	1	15	1.268	0.008
Lipoxygenase 7, chloroplastic	P38419	32.14	12	23	1.265	0.009
Cytochrome c	A2Y4S9	20.54	2	2	1.257	0.011
OSIGBa0106G07.1 protein	Q01IX2	15.81	6	7	1.253	0.012
Uncharacterized protein	A3A3Y3	15.32	4	4	1.249	0.013
Putative cytochrome P450	Q65X81	1.9	1	1	1.239	0.017
Os05g0301700 protein	Q0DJC3	36.18	1	6	1.238	0.018
Molybdopterin synthase catalytic subunit	A2X0R6	30.86	3	3	1.236	0.019
Os06g0320200 protein	A0A0P0WVX1	3.12	1	1	1.235	0.019
Long chain acyl-CoA synthetase	Q5W6W7	22.73	1	12	1.234	0.019
Putative uncharacterized protein	A2YFS4	12.05	3	6	1.229	0.022
Putative uncharacterized protein	B8AJE7	35.67	2	13	1.227	0.023
Putative uncharacterized protein	B8AQM5	30.27	1	4	1.224	0.025
Malate dehydrogenase	Q94JA2	50.29	6	15	1.220	0.027
Putative dihydroxypolyprenylbenzoate methyltransferase	Q5VMJ1	5.88	1	1	1.219	0.028
Malic enzyme	Q6T5D1	25.79	4	9	1.211	0.034
Uncharacterized protein	A3BUF9	1.66	1	1	1.209	0.035
Glutathione S-transferase	Q6WSC2	12.88	1	3	1.207	0.037
Hydroxycinnamoyltransferase 2	Q6K638	4.07	1	1	1.207	0.037
Putative uncharacterized protein	B8AQS4	2.69	1	1	1.206	0.038
Uncharacterized protein	A2Z9J2	8.23	2	2	1.204	0.040
Uncharacterized protein	A3C7L4	8.44	2	2	1.203	0.040
Beta-glucosidase 22	Q60DX8	15.2	6	7	1.202	0.041
Diacylglycerol kinase	Q6K4P5	18.85	8	8	1.202	0.042
Os01g0966000 protein	Q8LHD1	17.18	1	17	1.202	0.042
NADH-ubiquinone oxidoreductase chain 4	Q2F980	2.63	1	1	1.201	0.042
Chlorophyll a-b binding protein 1, chloroplastic	P12330	64.91	3	9	0.657	0.000
Putative uncharacterized protein	A2YLQ3	33.1	11	11	0.743	0.000
Uncharacterized protein	B9FUE0	7.43	1	1	0.756	0.001
Putative photosystem I chain V (Fragment)	Q710P6	12.16	2	2	0.772	0.002
Os06g0178900 protein	Q8H616	13.82	1	10	0.778	0.002
Flavonoid 3’-hydroxylase	Q7G602	4.56	1	1	0.779	0.002
Photosystem I iron-sulfur center	P0C359	82.72	7	7	0.781	0.003
Putative uncharacterized protein	A2YI66	6.77	1	1	0.782	0.003
Glutathione peroxidase	B7FAE9	30.34	1	6	0.787	0.003
Putative uncharacterized protein	B8ARH5	4.64	1	1	0.802	0.007
Putative uncharacterized protein	B8A9U7	11.48	1	5	0.807	0.008
Chlorophyll a-b binding protein, chloroplastic	A2YCB9	12.86	4	4	0.809	0.009
Uncharacterized protein	A3ACD7	15.98	1	11	0.822	0.016
Putative uncharacterized protein	B8A7Q2	4.45	2	2	0.825	0.018
Uncharacterized protein	A3BKU8	53.05	13	13	0.827	0.020
Fructose-bisphosphate aldolase, chloroplastic	Q40677	53.61	13	20	0.829	0.021
Putative uncharacterized protein	A2YKQ6	41.89	3	3	0.830	0.022
Photosystem II 10 kDa polypeptide	P93443	22.22	2	2	0.831	0.023

The differentially depressed proteins were devoted to energy metabolism, carbohydrate and lipid metabolism, nucleotide metabolism, amino acid metabolism, glycan biosynthesis and metabolism, terpenoids and polyketides metabolism, and other secondary metabolites biosynthesis, all of which belonged to C and N metabolism. The concerning differentially expressed proteins were listed in Tables 6 and 7.

## Discussion

This study found no significant difference in total biomass between T2A-1 and MH63 under different N treatments, indicating that the two lines had similar response to biomass assimilation. However, *Bt* rice line T2A-1 presented higher dry matter content in leaves and lower content in spike compared with its counterpart MH63. Previous studies reported other differences in agronomy traits. For example, lower yield caused by lower setting rates was also reported in several Bt rice lines introduced with a Cry2A*^31^, Cry1C*^20^, C*ry1Ab*^17^, *Cry1Ac*^14^, *Xa21*^4^ or *Bt/CpTI*^18^ gene. And shorter plant heights and root lengths^14^, fewer grains per panicle^17^ and lower setting rates^5,14,17,18,20^ have also been reported in recent years. These differences in agronomy traits could not be ignored for optimizing cultivation of transgenic crops.

Besides, the results of accumulation and distribution of N examination were similar to those of biomass in the two lines, T2A-1 and MH63. N content in spike of T2A-1 was lower than that in spike of MH63, potentially resulting in the weight loss of spike of T2A-1. The experiment with maize indicated that lower one in grains but higher N accumulation in stovers were found in several Bt hybrid maize strains, compared with their non-Bt counterparts^27^. And more N in kernels was detected in Pioneer 38W36Bt, a Bt hybrid maize, compared with the control Pioneer 38W36 (non-Bt), while N concentration and leaf chlorophyll content at the silking and maturity stages in Bt hybrid maize were similar to that in control group^28^. Bt cotton was reported to have more active N metabolism in the vegetative stage than its non-Bt counterpart, resulting in a reduction in boll size^29^. It could be concluded that there existed differences in N metabolism between Bt crops and their non-Bt crops including rice, maize and cotton.

Additionally, the significant differences in the accumulation of C between the two lines under several N treatments were also observed. But C distribution ratios in plant parts of the two lines were the same, i.e., 1:1:1. Proteomics analysis results revealed that the greatest difference in the number of differentially expressed proteins related to carbohydrate metabolism was observed between RN and N0 treatment. As we know, C metabolism provides material and energy for all metabolism processes in plant. Thus, changes on C metabolism would have enormous influence on plant growth and development. Our proteomics analysis results obtained by iTRAQ method exhibited that differentially expressed proteins mainly functioned in energy metabolism, carbohydrate metabolism, and so on. Previous reports on Bt cotton revealed that differentially expressed proteins identified between transgenic *Bt* + *CpTI* cotton SGK321 and its counterpart SY321 accounted for approximate 10% of all proteins identified by using proteomics analysis. These differentially expressed proteins mainly involved in carbon fixation and photosynthesis, glyoxylate and dicarboxylate metabolism pathway, oxidative pentose phosphate pathway^32^. 2-D PAGE experiment by Ren *et al*. (2009) reported the detection of 102 differentially expressed proteins in 12 transgenic Arabidopsis, compared with their wild-type (ecotype Col-o)^33^. Their experiment also reported that most of these differentially expressed proteins were involved in energy transfer, oxidative respiration, and photosynthesis. In addition, *Gong et al*. used comparative proteomics approaches to determine proteome differences in seeds between 2 transgenic rice lines and their corresponding control groups, namely, D68 and MH86^34^. Mass spectrometry analysis exhibited that differentially expressed proteins participated in several cellular and metabolic processes, including protein synthesis and transportation, and defense response. And differentially expressed proteins were also detected in seeds of transgenic and non-transgenic soybean by comparative proteomics approach^35^. Therefore, it can be concluded that changes in C metabolism were also widely found in transgenic plants.

It is well known that C metabolism and N metabolism are always intertwined in plant^36^. Up to 55% of net plant carbon was devoted to nitrogen assimilation and metabolism in some tissues^37^. Carbon and nitrogen metabolism are intimately linked. This study revealed different C/N ratios in leaf between T2A-1 and MH63. The C/N ratio in whole aboveground part of T2A-1 was significantly lower than that of MH63. C/N ratio has influence on glucose metabolism, assimilate transportation, senescence process of leaf, etc. Thus, as plant material and energy suppliers, C and N metabolism ought to be taken into consideration simultaneously in their function assessment.

Until now, there were three hypotheses of unintended effects brought by transgenic technology. But insertion position of transgenic rice line T2A-1 used in our study was noncoding region, and it was chosen to exhibit the best field resistance and excellent agronomy traits by several field experiment among 102 transgenic rice lines^6^. Thus, we are inclined to attribute these effects to bio-burden caused by additional Bt protein synthesis. As a soluble protein, Bt protein would consume extra N and energy in Bt crops. And C and N metabolism were assumed as basic processes regulating N allocation and energy distribution in plant. Thus, it has drawn a wide concern whether the *Bt* protein synthesis process will influence the fundamental C and N metabolism in *Bt* crops resulting in a possible “bio-burden” affecting plant growth and development^18,24,38,39^. Poor adaptabilities to nutrient deficiency and several abiotic stresses were reported in some *Bt* cotton lines in previous studies. For example, experiment conducted by *Wei et al*. revealed that root activity (less root organic acid exudation) in several Bt cotton lines under low N supply condition was lower than that in their non-Bt wild types^40^. And lower biomass was also observed in some Bt cotton lines under low K application condition than their non-Bt counterparts^41^. Besides, some Bt cotton lines were reported to have suffered more from pathogens stress^42^ and CeO_2_ nanoparticles stress^43^ than their non-Bt wild types. Another experiment by Li *et al*. (2015) indicated that Bt cottons adapted poorly to drought stress or elevated O_3_ stress^42^. And inhibited nitrogen metabolism by salinity, waterlogging and the combined stress was observed to result in the decline of Bt protein expression, causing bollworm control reduction^44^.

Based on the discussion above, it could be concluded that the phenotypic and yield changes of Bt crops under different conditions were frequently occurring phenomena. However, the mechanism of such changes has not been known well until now. Our study revealed that differences in C and N partitioning could be one of possible reasons accounting for changes in yield and phenotypic traits in T2A-1. Future study could make a further exploration of energy utilization related to C and N metabolism under the framework of the “bio-burden” hypothesis comparing more transgenic plants and their wild types.

## Materials and Methods

### Materials

Bt rice line T2A-1, expressing Cry2A* protein, was used for the assessment of C and N metabolism processes in the plant. And its conventional (non-Bt) parent line MH63, an excellent three-line rice restorer line, served as a control in this study. Both of the two rice lines were provided by National Key Laboratory of Crop Genetic Improvement, Huazhong Agricultural University. During the transformation process, a series of Cry2A* rice lines were obtained from the modification of the Cry2A* gene with no amino acid sequence increased. Besides, the insertion position of Cry2A* in T2A-1 was a noncoding region, which theoretically had few effects on exogenous gene expression. And T2A-1 was found to exhibit the best field resistance and excellent agronomy traits, therefore it was selected for the experiment among 102 independent transformants obtained^6^.

### Soils

Considering its low nitrogen content, loess soil was chosen for this nitrogen concentration gradient experiment at the Agricultural Experiment Station of Huazhong Agriculture University, Wuhan, China. The soil was first air-dried in open air environment at normal temperature, and then ground, finally mixed with sand before use. The soil contained 18.48 g kg^−1^ of organic matter, 0.11 g kg^−1^ of total N, and 1.19 g kg^−1^ of C with a pH being 6.56.

### Experimental Design

The nitrogen gradient experiment using T2A-1 and MH63 was carried out from May to September 2016 in net house. Four nitrogen fertilizer treatments were performed in this study, namely, supplying 0.2 g (N1 treatment), 0.35 g (N2 treatment), 0.5 g (N3 treatment), and 1 g (N4 treatment) nitrogen, respectively, in the whole growing period of rice. Nitrogen fertilizer in form of urea was applied three times during the whole developing period with applying ratio being 50% as base fertilizer, 30% at mid-tillering stage, and 20% at young panicle differentiation stage, respectively. In addition, potassium and phosphorus fertilizers were applied in a common way. Seedlings were transplanted to PVC pipes (16 cm × 55 cm). One seedling was planted into each pipe, then three pipes were placed into one bucket as a group. And there were three buckets as three replicates for each nitrogen fertilizer treatment using T2A-1 and MH63. These treatments were arranged in a completely random arrangement. One whole plant would be sampled and separated into leaf, stem and spike part for C and N content measurement at each sampling time. And two sampling times were set during reproductive periods including the flowering stage (FS), and 15 days after the flowering, named as FS, and 15 d after FS, respectively.

In addition, to explore the different expression of proteins related to C and N metabolism between MH63 and T2A-1, the other experiment concluding two nitrogen treatments was conducted for proteomics detection at the same time in the same screen house. And 0 g (N0 treatment) and 1 g (RN treatment) nitrogen fertilizer application treatments during the whole rice growth period were chosen to enlarge the difference between the two lines. Flag leaves were collected on day 15 after the flowering for proteomics testing.

### Total Carbon and Nitrogen Measurement

Leaf, stem and spike parts were separated from one sampling plant. All samples were oven-dried at 70 °C to constant weight before testing, and the final content of C and N was obtained through the formula that content percentage measured by elemental analyzer (Vario ISOTOPE, Elementar) multiplied dry weight of each sample. Nitrogen accumulation equated to the sum of N content of leaf, stem with sheath, and spike.

### Procedure of iTRAQ (isobaric tags for relative and absolute quantifica)Protein Extraction

The samples were frozen in liquid nitrogen and ground with pestle and mortar. Five times volume of TCA/acetone (1:9) was added to the powder and mixed by vortex. The mixture was placed at −20 °C for 4 h, and centrifuged at 6000 g for 40 min at 4 °C. The supernatant was discarded. The pre-cooling acetone was added and washed for three times. The precipitate was air dried. Then, 30 times volume of SDT buffer was added to 20–30 mg powder. After mixing and 5 min boiling, the lysate was sonicated and then boiled for another 15 min. Followed by being centrifuged at 14000 g for 40 min, the supernatant was filtered with 0.22 µm filters. The filtrate was quantified with the BCA Protein Assay Kit (Bio-Rad, USA). The sample was stored at −80 °C.

### Protein preparation, digestion and iTRAQ labelling

200 μg of proteins from each sample were incorporated into 30 μl SDT buffer (4% SDS, 100 mM DTT, 150 mM Tris-HCl pH 8.0). The detergent, DTT, and other low-molecular-weight components were removed by using UA buffer (8 M Urea, 150 mM Tris-HCl pH 8.0) through repeated ultrafiltration (Microcon units, 10 kD). Then 100 μl of iodoacetamide (100 mM IAA in UA buffer) was added to block reduced cysteine residues and the samples were incubated for 30 min in darkness. The filters were washed first with 100 μl UA buffer three times and then with 100 μl of Dissolution buffer (DS buffer) twice. Finally, the protein suspensions were digested with 4 μg of trypsin (Promega) in 40 μl of DS buffer overnight at 37 °C, and the obtained peptides were collected as a filtrate. The peptides from each sample were desalted on C18 Cartridges (Empore™ SPE Cartridges C18 (standard density), bed I.D. 7 mm, volume 3 ml, Sigma), concentrated by vacuum centrifugation and reconstituted in 40 µl of 0.1% (v/v) formic acid. The peptide content was estimated by UV light spectral density at 280 nm using an extinctions coefficient of 1.1 of 0.1% (g/l) solution that was calculated on the basis of the frequency of tryptophan and tyrosine in vertebrate proteins.

An eight-plex iTRAQ was set for the proteomics analysis, including N and N0 (Table 8). And 1 and 2 represented the replicates. Each replicate entailed 4 biological sampling replicates. 100 μg of peptide mixture from each sample was labelled by using iTRAQ reagent according to the manufacturer’s instructions (Applied Biosystems).

**Table 8:** Sample set of quantitative proteomic analysis.

iTRAQ label	113	114	115	116	117	118	119	121
samples	1 N-T2A-1	2 N-T2A-1	1 N0-T2A-1	2 N0-T2A-1	1 N-MH63	2 N-MH63	1 N0-MH63	2 N0-MH63

### Peptide Fractionation with Strong Cation Exchange (SCX) Chromatography

iTRAQ-labelled peptides were fractionated by SCX chromatography using the AKTA Purifier system (GE Healthcare). The dried peptide mixture was reconstituted and acidified with buffer A (10 mM KH_2_PO_4_ in 25% of ACN, pH 3.0) and loaded onto a PolySULFOETHYL 4.6 × 100 mm column (5 µm, 200 Å, PolyLC Inc, Maryland, U.S.A.). The peptides were eluted at a flow rate of 1 ml/min with a gradient of 0–8% buffer B (500 mM KCl, 10 mM KH2PO4 in 25% of ACN, pH 3.0) for 22 min, 8–52% buffer B from 22 min to 47 min, 52–100% buffer B from 47 min to 50 min, 100% buffer B from 50 min to 58 min, and buffer B was reset to 0% after 58 min. The elution was monitored by absorbance at 214 nm, and fractions were collected every 1 min. The collected fractions were desalted on C18 Cartridges (Empore™ SPE Cartridges C18 (standard density), bed I.D. 7 mm, volume 3 ml, Sigma) and concentrated by vacuum centrifugation.

### LC-MS/MS Analysis by Q Exactive

LC-MS/MS analysis was performed on a Q Exactive mass spectrometer (Thermo Scientific) that was coupled to Easy nLC (Proxeon Biosystems, now Thermo Fisher Scientific) for 60 min. The mass spectrometer was operated in positive ion mode. MS data was acquired by using a data-dependent top10 method dynamically choosing the most abundant precursor ions from the survey scan (300–1800 m/z) for HCD fragmentation. Automatic gain control (AGC) target was set to 3e6, and maximum inject time to 10 ms. Dynamic exclusion duration was 40.0 s. Survey scans were acquired at a resolution of 70,000 at m/z 200 and the resolution for HCD spectra was set to 17,500 at m/z 200, and isolation width was 2 m/z. Normalized collision energy was 30 eV and the underfill ratio, which specifies the minimum percentage of the target value likely to be reached at maximum fill time, was defined as 0.1%. The instrument was run with peptide recognition mode enabled.

### Data analysis

SAS 9.1 software (SAS Institute, Inc., Cary, NC, USA) was used for the analysis of variance (ANOVA) of data referring to C and N distribution in this experiment. And these data are expressed as the means ± standard deviation (SD, n = 3). Differences of the means were statistically significant at *α* = *0*.*05*.

Besides, to conduct proteomics analysis, MS/MS spectra were searched using MASCOT engine (Matrix Science, London, UK; version 2.2) embedded into Proteome Discoverer 1.4. Parameters were set as shown in Table 9. Database of uniprot _Oryza_sativa_168274 _ 20170123.fasta (http://www.uniprot.org) was used for proteomics analysis. All data related to this study has been public available on iProX (www.iprox.com) with ID IPX0001090000 (http://www.iprox.org/page/PDV014.html?projectId=IPX0001090000).

**Table 9:** Parameters setting of MASCOT engine.

Item	Value
Enzyme	Trypsin
Max Missed Cleavages	2
Fixed modifications	Carbamidomethyl (C), iTRAQ 8plex (N-term), iTRAQ 8plex (K)
Variable modifications	Oxidation (M), iTRAQ 8plex (Y)
Peptide Mass Tolerance	±20 ppm
Fragment Mass Tolerance	0.1 Da
Database	uniport _Oryza_sativa_168274 _ 20170123.fasta
Database pattern	Decoy
Peptide FDR	≤0.01
Protein Quantification	The protein ratios are calculated as the median of only unique peptides of the protein
Experimental Bias	Normalizes all peptide ratios by the median protein ratio. The median protein ratio should be 1 after the normalization.
